# LncRNA MALAT1-deficiency restrains lipopolysaccharide (LPS)-induced pyroptotic cell death and inflammation in HK-2 cells by releasing microRNA-135b-5p

**DOI:** 10.1080/0886022X.2021.1974037

**Published:** 2021-09-09

**Authors:** Jie Huang, Chen Xu

**Affiliations:** Wuhan Fourth Hospital, Puai Hospital, Tongji Medical College, Huazhong University of Science and Technology, Hubei, PR China

**Keywords:** Chronic kidney disease, NLRP3-mediated cell pyroptosis, LncRNA MALAT1, microRNA-135b-5p, cell apoptosis

## Abstract

Long non-coding RNAs (LncRNAs) participate in the regulation of chronic kidney disease (CKD), and acute kidney injury (AKI) is identified as an important risk factor for CKD. This study investigated the involvement of a novel LncRNA MALAT1 in regulating lipopolysaccharide (LPS)-induced cell pyroptosis and inflammation in the human renal tubular epithelial HK-2 cells. Here, the HK-2 cells were subjected to LPS (2 μg/mL) treatment to establish cellular AKI models *in vitro*, and we validated that LPS triggered NLRP3-mediated pyroptotic cell death, promoted cell apoptosis and inflammation-associated cytokines secretion to induce HK-2 cell injury. Then, a novel LncRNA MALAT1/miRNA (miRNA)-135b-5p axis was verified to rescue cell viability in LPS treated HK-2 cells by targeting NLRP3. Mechanistically, miRNA-135b-5p bound to LncRNA MALAT1, and LncRNA MALAT1 positively regulated NLRP3 through acting as RNA sponger for miRNA-135b-5p. Further gain- and loss-of-function experiments evidenced that both LncRNA MALAT1 ablation and miRNA-135b-5p overexpression reversed LPS-induced cell pyroptosis, apoptosis, and inflammation in the HK-2 cells, and the protective effects of LncRNA MALAT1 knock-down on LPS-treated HK-2 cells were abrogated by silencing miRNA-135b-5p. In general, our study firstly investigated the role of the LncRNA MALAT1/ miRNA-135b-5p/NLRP3 signaling cascade in regulating LPS-induced inflammatory death in HK-2 cells.

## Introduction

Chronic kidney disease (CKD) damages normal kidney functions and brings huge health burden for human beings worldwide, and about 8–16% patients are annually diagnosed with CKD [1]. Of note, as the results of its complicated pathogenesis, the therapeutic efficacy of the current strategies for CKD treatment is seriously limited [2,3]. In addition, previous literatures identify that acute kidney injury (AKI) is the main risk factor for CKD [4], and transition from AKI to CKD seriously degrades the life quality of human beings [5]. In recent publications, researchers agree that cell pyroptosis-mediated super-inflammation is pivotal for the pathogenesis of AKI-associated CKD [6,7]. Therefore, it is worthwhile to investigate whether elimination of cell pyroptosis and inflammation is effective to cure AKI-associated CKD, and more diagnostic and therapeutic biomarkers need to be verified. Thus, to explore this issue, the human renal tubular epithelial HK-2 cells were subjected to lipopolysaccharide (LPS) treatment to induce cellular AKI models *in vitro* as previously described [8,9].

Long non-coding RNAs (LncRNAs) are recently identified as key regulators for various diseases, such as cancers [10–12] and liver fibrosis [13,14]. Interestingly, data from Li et al. [15] and Santer et al. [16] evidence that LncRNAs are validated as potential prognostic biomarkers for CKD, but the information regarding to this field is still scarce, which makes it urgent and necessary to explore the relationship between LncRNAs and CKD progression. Interestingly, LPS alters the expression patterns of multiple LncRNAs to facilitate the development of Alzheimer’s disease [17], chondrocytes injury [18], and sepsis [19], and pyroptotic pathways mediated cell death can be regulated by LncRNAs [20–22]. Based on the above information, our preliminary work screens out a novel LncRNA MALAT1 that can be positively regulated by LPS treatment in the HK-2 cells. As previously described, LncRNA MALAT1 positively regulates inflammation [23–25] and previous work suggests that upregulation of LncRNA MALAT1 aggravates NLRP3-mediated pyroptotic cell death in multiple diseases, including diabetic atherosclerosis [26] and diabetic nephropathy [27]. Of note, Song et al. [21] and Liu et al. [28] evidence that silencing of LncRNA MALAT1 is effective to protect against high-glucose-induced endothelial and epithelial cell pyroptosis.

MicroRNAs (miRNAs) function as ‘bridge’ to combine LncRNAs and mRNA [29,30], and various LncRNAs-miRNAs-mRNA competing for endogenous RNA (ceRNA) networks are identified to be closely associated with CKD pathogenesis [8,31]. In addition, LncRNA MALAT1 exerts its inflammation-promoting effects through modulating miR-181c-5p [32], miR-26b [33], and miR-590 [34], and Dai et al. notice that knock-down of LncRNA MALAT1 releases miR-146a to ameliorate LPS-induced acute lung injury [35], and LncRNA MALAT1 regulates LPS-induced cytokines secretions in human gingival fibroblasts by sponging miR-20a [36]. Among all the miRNAs, miRNA-135b-5p targets both LncRNA MALAT1 [37] and 3′ untranslated regions of NLRP3 mRNA [38,39], and Li et al. evidence that miRNA-135b-5p silences AMPK phosphatase Ppm1e to restrain LPS-mediated production of pro-inflammatory cytokines [40]. In addition, miRNA-135b-5p is reported to be relevant to renal fibrosis [41].

Herein, this study showed that LPS triggered pyroptotic cell death and inflammatory injuries in HK-2 cells *in vitro*. Moreover, it was revealed that the LncRNA MALAT1/miRNA-135b-5p/NLRP3 signaling cascade could be modulated by LPS treatment, and targeting this pathway was effective to rescue cell viability in the HK-2 cells treated with LPS. Our preliminary data provided new biomarkers for CKD diagnosis and therapy, which also broadened our knowledge in this field.

## Materials and methods

### Cell culture, treatment, and vectors transfection

Human renal tubular epithelial HK-2 cells were bought from American Type Culture Collection (ATCC, Manassas, VA) and were maintained in the DMEM/F12 medium (Gibco, Waltham, MA) containing 10% FBS (Gibco, Waltham, MA) under the standard culture conditions with 5% CO_2_ at 37 °C. Based on our preliminary work (data not shown), the HK-2 cells at passage 3–6 were subjected to LPS (2 μg/mL) treatment for 0, 24, and 48 h, respectively. In addition, the gene manipulating vectors for LncRNA MALAT1, and miRNA-135b-5p mimic and inhibitor were constructed by Sangon Biotech (Shanghai, China) according to the previous publications [37–39].

### Real-time qPCR

Following the protocols provided by the previous studies [37–39], the real-time qPCR was performed to examine genes expression levels at transcriptional levels. Briefly, total RNA was obtained by TRIzol (Beyotime, Shanghai, China) and analyzed by the following agarose electrophoresis. Next, the total RNA was reversely transcribed, and the quantification of the target genes were measured by using the SYBR Green PCR kit (Qiagen, Hilden, Germany) . The primer sequences could be found in the previous publications [6,7,37–39].

### Western blot

RIPA lysis buffer (Beyotime, Shanghai, China) was bought to extract total proteins from the cells, which were further separated by using the 10% SDS-PAGE. Then, according to the molecular weight of the proteins, the target proteins were transferred onto the polyvinylidene fluoride (PVDF, Solarbio, Beijing, China) membranes. The PVDF membranes with transferred proteins were subsequently blocked by 5% blocking buffer and were probed with the primary antibodies against NLRP3 (1:1500, Abcam, Cambridge), ASC (1:2000, Abcam, Cambridge), IL-1β (1:2000, Takara, Berkeley, CA), IL-18 (1:1500, Takara, Berkeley, CA) and GAPDH (1:2000, Abcam, Cambridge) overnight at 4 °C and were incubated with the secondary antibodies for 1 h at room temperature. Finally, the protein bands were visualized by the ChemiDoc^TM^ XRS system (Bio-Rad, Hercules, CA) and GAPDH was used as internal control. The Image J software was employed to analyze the gray values of the protein bands.

### Cell proliferation and viability

The HK-2 cells were seeded onto the 96-well plates at the density of 5 × 10^4^ cells per well and were subjected to LPS treatment for 0, 24, and 48 h, respectively. The cells were then incubated with the MTT solution (20 μL/well) for 1 h at 37 °C, which were vortexed and a microplate reader (Bio-Rad, Hercules, CA) was purchased to examine the OD values to reflect relative cell proliferation. Moreover, the LPS treated HK-2 cells were stained with trypan blue staining buffer for 20 min at 37 °C, and a microscope (ThermoFisher Scientific, Waltham, MA) was used to observe and count the dead blue cell numbers, which were used for calculating cell viability.

### Enzyme-linked immunosorbent assay (ELISA)

The HK-2 cells were subjected to LPS treatment for 48 h, and the supernatants were collected and analyzed by using the commercial ELISA kit (Solarbio, USA) in keeping with their protocols. The relative expressions of the inflammatory cytokines (IL-1β, IL-18, IL-6, and TGF-β) were measured by using a microplate reader (Bio-Rad, Hercules, CA) at the wavelength of 450 nm.

### Examination of cell apoptosis

The HK-2 cells were treated with LPS for 48 h, and the cells were stained with the Annexin V-FITC and PI dyes for 40 min at room temperature without light exposure according to the protocols of Cell Apoptosis Detection Kit (Invitrogen, Waltham, MA). Then, a flow cytometer (Becton Dickinson, Franklin Lakes, NJ) was used to examine cell apoptosis ratio, the cells stained with either Annexin V-FITC or PI were regarded as dead cells.

### Dual-luciferase reporter gene system assay

The binding sites in LncRNA MALAT1 and miRNA-135b-5p were predicted by using the online starBase software (http://starbase.sysu.edu.cn/), which were validated by the following dual-luciferase reporter gene system assay in keeping with the previous work [8,31]. The wild-type and mutant LncRNA MALAT1 were cloned into luciferase reporters by Sangon Biotech (Shanghai, China), and were co-transfected with the miRNA-135b-5p mimic into the HK-2 cells.

### Data analysis

The data in this study were collected and were shown as means ± standard deviation (SD), which were further analyzed by SPSS version 18.0 software (SPSS Inc., Chicago, IL). Specifically, the Student’s t-test was used to compare the means from two groups, and the comparisons among multiple groups were conducted by using the one-way ANOVA analysis. Individual experiment had three times of biological repetitions, and each biological replicate contained three times of technical replicates. *p < .05 was regarded as statistical significance.

## Results

### LPS treatment significantly influenced cellular functions and promoted inflammatory injury in HK-2 cells

**Figure 1.**
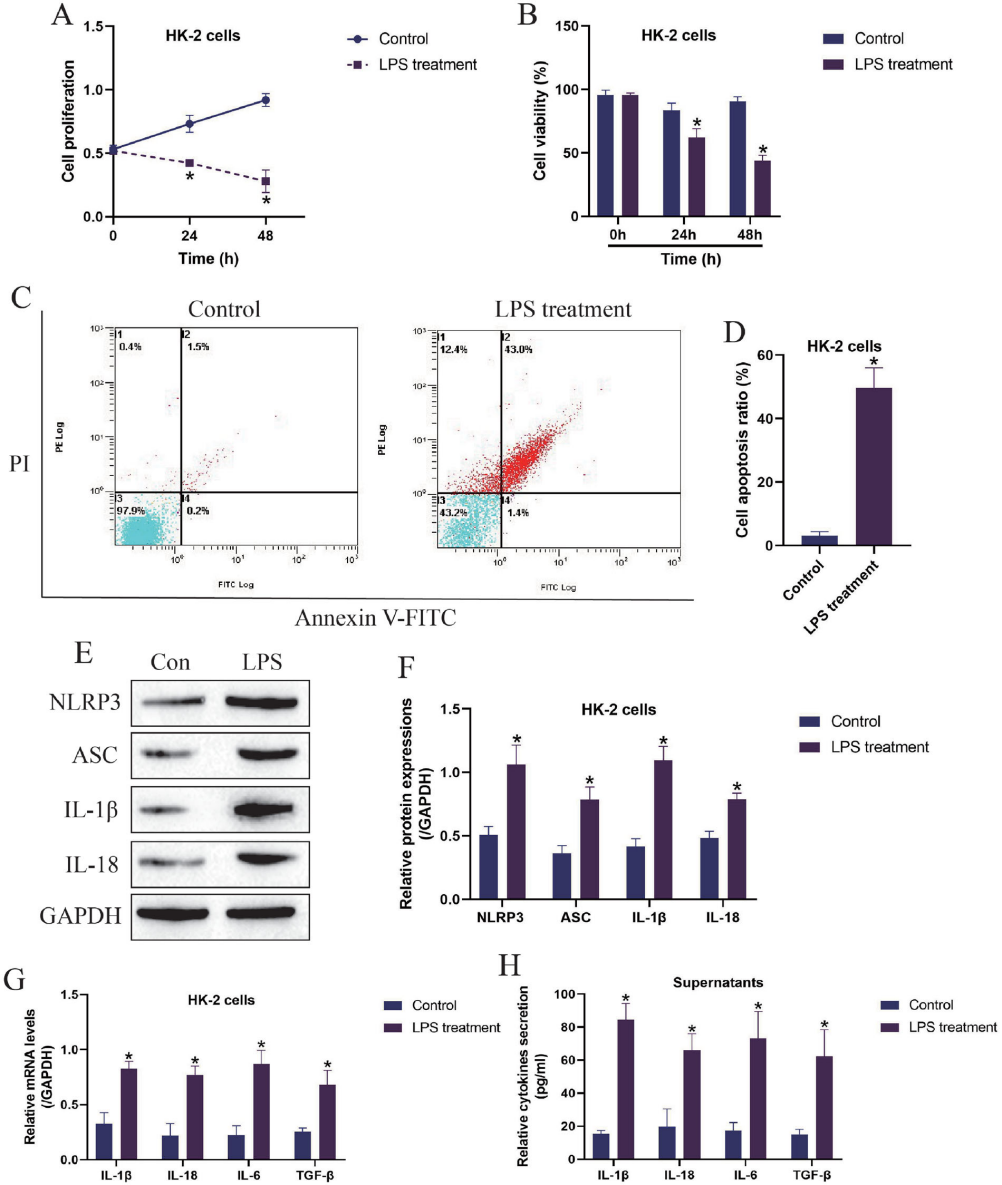
LPS triggered pyroptotic cell death and inflammatory responses in HK-2 cells. (A) Cell proliferation and (B) viability were examined by MTT assay and trypan blue staining assay. (C,D) The HK-2 cells were stained with Annexin V-FITC and PI, and the apoptotic cell ratio was determined by flow cytometer. (E,F) The pyroptosis-associated biomarkers in HK-2 cells were measured by Western Blot analysis. The uncropped WB images are shown in Figure S1. (G) Real-time qPCR and (H) ELISA were performed to evaluate inflammatory cytokines generation and secretion in HK-2 cells and its supernatants. Individual experiment had three times of biological repetitions, and each biological replicate contained three times of technical replicates, and *p < .05.

Based on the experimental protocols provided by the previous work [8] and our preliminary experiments (data not shown), the HK-2 cells were exposed to 2 μg/mL of LPS for 0, 24, and 48 h to establish the nephritic injury cell models. As shown in Figure 1(A), the MTT assay results showed that LPS suppressed cell proliferation abilities in a time-dependent manner, and consistent with this, the trypan blue staining assay results in Figure 1(B) supported that HK-2 cell viability were also significantly inhibited by LPS treatment. Next, the HK-2 cells with or without LPS stimulation were stained with Annexin V-FITC and PI, and the following flow cytometer results showed that LPS significantly increased both apoptotic and necroptotic cell ratio in the HK-2 cells (Figure 1(C,D)). In addition, by performing Western Blot analysis, we noticed that the protein levels of cell pyroptosis associated biomarkers, including NLRP3, ASC, IL-1β, and IL-18 were apparently upregulated by LPS (Figure 1(E,F)), indicating that LPS triggered pyroptotic cell death in HK-2 cells. Moreover, the supernatants of the HK-2 cells were collected, and the Real-Time qPCR results in Figure 1(G) and the ELISA results in Figure 1(H) evidenced that LPS promoted pro-inflammatory cytokines (IL-1β, IL-18, IL-6, and TGF-β) generation and secretion in HK-2 cells and its supernatants.

### The regulating mechanisms of LncRNA MALAT1, microRNA-135b-5p, and NLRP3 in HK-2 cells

**Figure 2.**
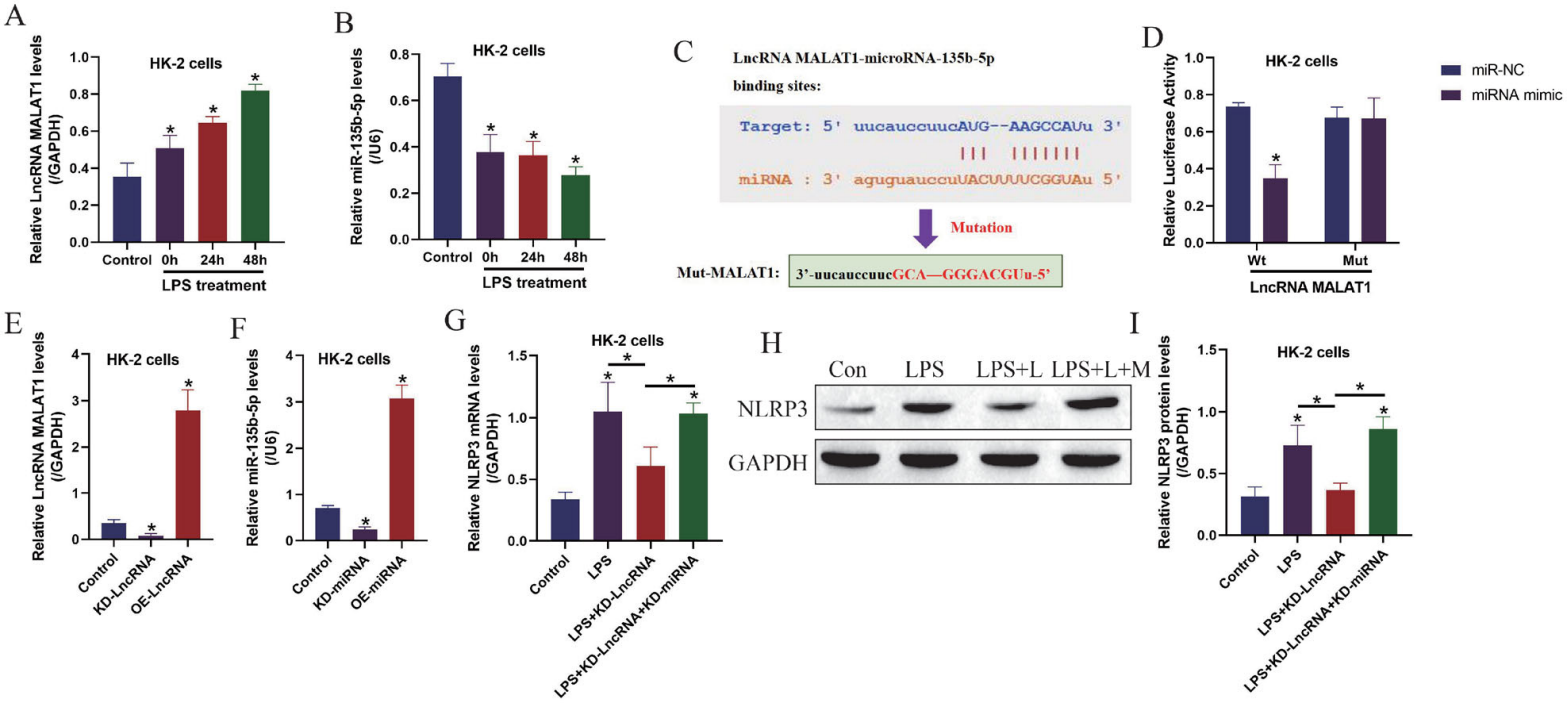
The regulating effects of LPS treatment on the LncRNA MALAT1/microRNA-135b-5p axis. The expression levels of (A) LncRNA MALAT1 and (B) microRNA-135b-5p were detected by Real-Time qPCR. (C) Prediction and (D) validation of the binding sites in LncRNA MALAT1 and microRNA-135b-5p. (E,F) The LncRNA MALAT1/microRNA-135b-5p axis was manipulated in HK-2 cells, and the vectors transfection efficacy was examined by Real-Time qPCR. (G–I) LPS promoted NLRP3 expressions in HK-2 cells by regulating the LncRNA MALAT1/microRNA-135b-5p axis. The uncropped WB images are shown in Figure S2. Individual experiment had three times of biological repetitions, and each biological replicate contained three times of technical replicates, and *p < .05.

Given that LncRNAs/ miRNAs ceRNA networks are closely associated with CKD pathogenesis and LPS-induced inflammatory injury, which encouraged us to investigate this issue, and here we identified that the expression levels of LncRNA MALAT1 and miRNA-135b-5p could be regulated by LPS treatment (Figure 2(A–I)). Specifically, LPS significantly upregulated LncRNA MALAT1 (Figure 2(A)), while downregulated miRNA-135b-5p in HK-2 cells (Figure 2(B)). Interestingly, through conducting bioinformatics analysis, we noticed that miRNA-135b-5p potentially bound to LncRNA MALAT1 (Figure 2(C)), which were supported by the following dual-luciferase reporter gene system assay (Figure 2(D)). As shown in Figure 2(D), miRNA-135b-5p mimic significantly decreased relative luciferase activities in the HEK293T cells co-transfecting with wild-type LncRNA MALAT1. In addition, LncRNA MALAT1 (Figure 2(E)) and miRNA-135b-5p (Figure 2(F)) were respective overexpressed and silenced in HK-2 cells, and we found that knock-down of LncRNA MALAT1 decreased NLRP3 mRNA (Figure 2(G)) and protein (Figure 2(H,I)) levels in the LPS treated HK-2 cells, which were reversed by silencing miRNA-135b-5p, suggesting that LncRNA MALAT1 exerted its regulating effects on NLRP3 by releasing miRNA-135b-5p.

### Both LncRNA MALAT1 and microRNA-135b-5p involved in regulating LPS-induced HK-2 cell pyroptosis

**Figure 3.**
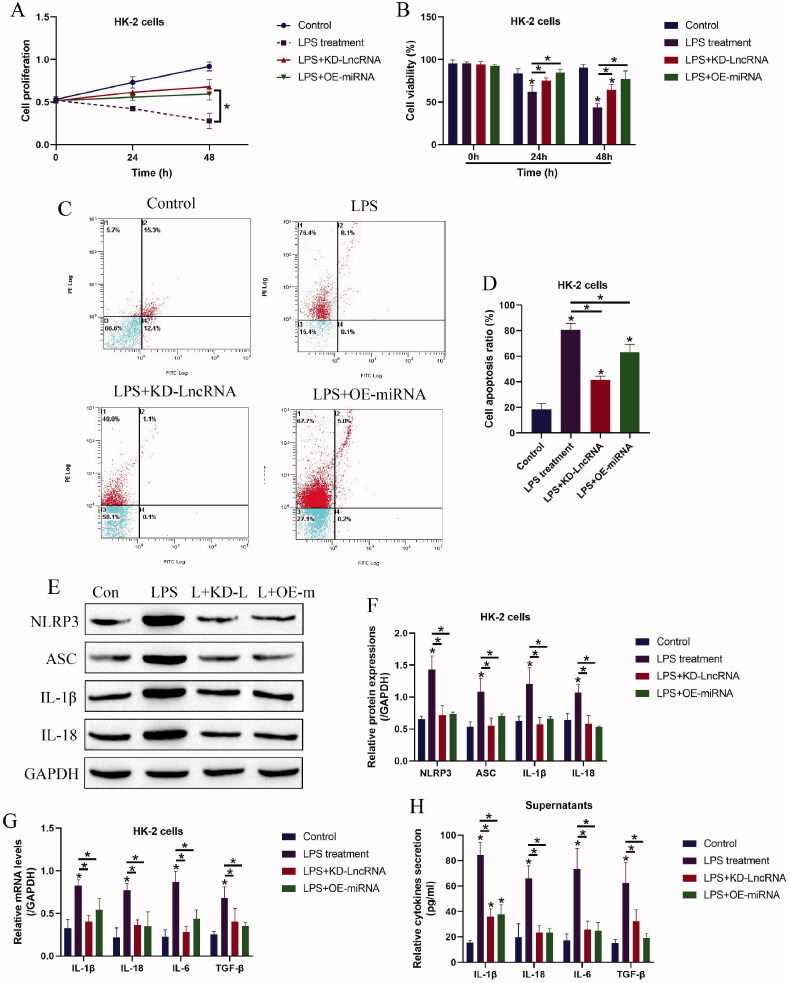
LPS triggered inflammatory cell death in HK-2 cells by modulating LncRNA MALAT1 and microRNA-135b-5p. Examination of (A) cell proliferation and (B) viability by MTT assay and trypan blue staining assay. (C,D) Annexin V-FITC and PI double staining assay was utilized to determine cell apoptosis ratio in HK-2 cells. (E,F) Western Blot analysis was performed to measure the expression levels of NLRP3, ASC, IL-1β, and IL-18 in HK-2 cells. The uncropped WB images are shown in Figure S3. (G) Real-Time qPCR and (H) ELISA were respectively performed to examine production and secretion of the inflammation-associated cytokines in HK-2 cells and its supernatants. Individual experiment had three times of biological repetitions, and each biological replicate contained three times of technical replicates, and *p < .05.

Since both LncRNA MALAT1 and miRNA-135b-5p can be modulated by LPS treatment, we conjectured that the above two genes might be closely associated with LPS-induced cell death and inflammation in HK-2 cells. As shown in Figure 3(A,B), LncRNA MALAT1 ablation and miRNA-135b-5p overexpression rescued cell proliferation and viability in HK-2 cells treated with LPS. Then, the following data in Figure 3(C,D) supported that LPS-induced cell apoptosis in HK-2 cells was also suppressed by silencing LncRNA MALAT1 and upregulating miRNA-135b-5p. In addition, the pyroptosis-associated biomarkers were examined by Western Blot analysis, which showed that the promoting effects of LPS treatment on NLRP3, ASC, IL-1β, and IL-18 were abrogated by both LncRNA MALAT1 ablation and miRNA-135b-5p overexpression (Figure 3(E,F)). Consistently, the real-time qPCR results in Figure 3(G) and the ELISA in Figure 3(H) suggested that LPS-induced cytokines generation and secretions, including IL-1β, IL-18, IL-6, and TGF-β, were also hampered by silencing LncRNA MALAT1 and upregulating miRNA-135b-5p.

### Knock-down of LncRNA MALAT1 reversed LPS-induced cell death in HK-2 cells by releasing microRNA-135b-5p

**Figure 4.**
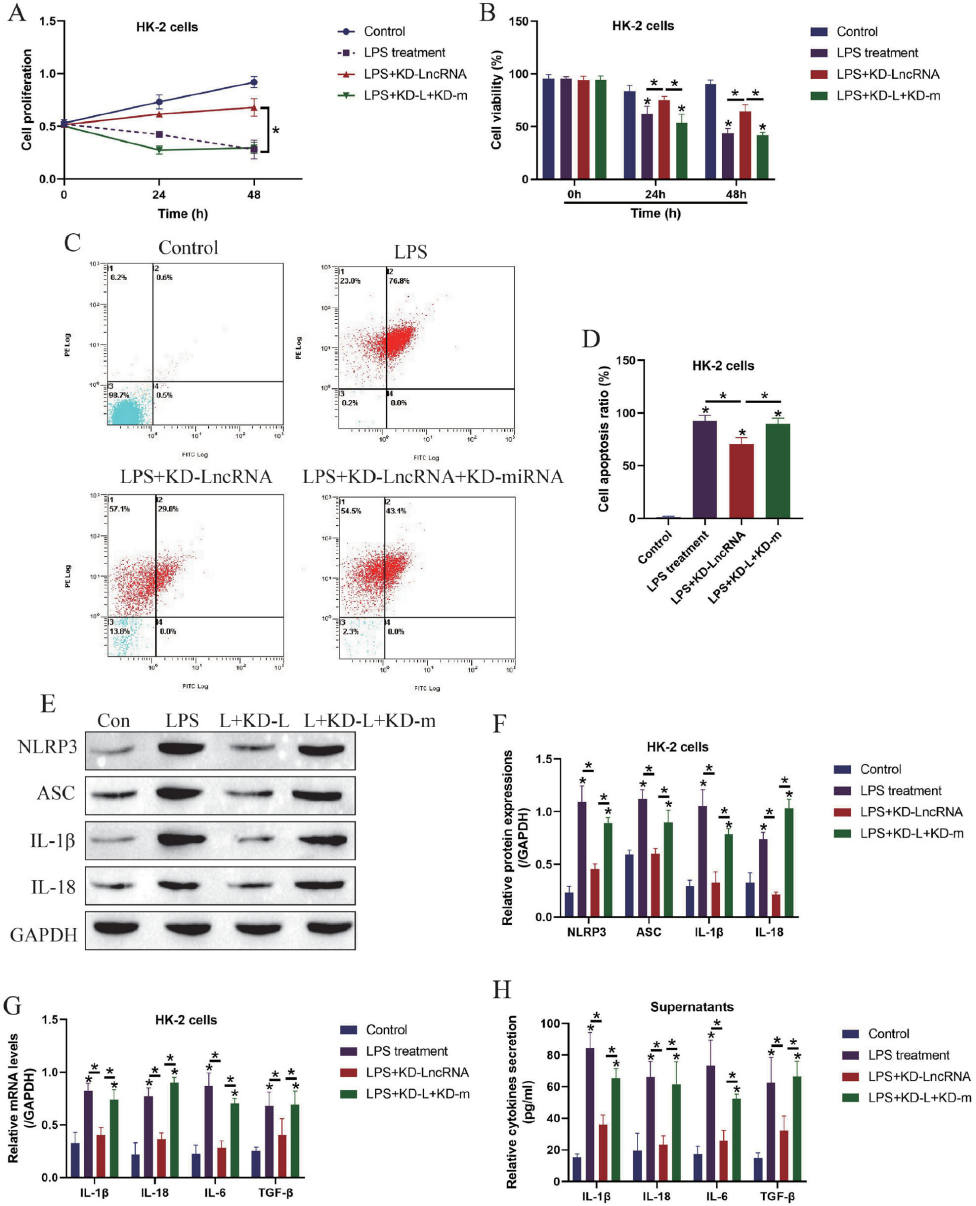
Knock-down of LncRNA MALAT1 attenuated LPS-induced cell pyroptosis in HK-2 cells by releasing microRNA-135b-5p. (A) Cell proliferation and (B) viability were determined by MTT assay and trypan blue staining assay. (C,D) Cell apoptosis ratio in HK-2 cells was examined. (E,F) Western Blot analysis was performed to examine the levels of pyroptosis-associated proteins. The uncropped WB images are shown in Figure S4. (G,H) The levels of inflammation-associated cytokines were determined in HK-2 cells and its supernatants. Individual experiment had three times of biological repetitions, and each biological replicate contained three times of technical replicates, and *p < .05.

We next investigated whether targeting LncRNA MALAT1 ameliorated LPS-induced pyroptotic cell death in HK-2 cells by releasing miRNA-135b-5p, and to achieve this, the LncRNA MALAT1 downregulation vectors and miRNA-135b-5p inhibitor were co-transfected into the HK-2 cells (Figure 2(E,F)), which were subsequently treated with LPS stimulation. The HK-2 cells were divided into three groups, including control, LncRNA MALAT1 silencing (KD-LncRNA MALAT1), LncRNA MALAT1 silencing plus miRNA-135b-5p knock-down (KD-LncRNA MALAT1 + KD-miRNA), and the functional experiments were performed. As expected, silencing of LncRNA MALAT1 rescued cell proliferation (Figure 4(A)) and viability (Figure 4(B)) in LPS treated HK-2 cells, which were reversed by miRNA-135b-5p inhibitor. Similarly, the Annexin V-FITC- and PI-positive apoptotic cell ratio in the LPS-treated HK-2 cells was decreased by LncRNA MALAT1 ablation, which were also abrogated by miRNA-135b-5p silence (Figure 4(C,D)). In addition, the LncRNA MALAT1/ miRNA-135b-5p axis regulated cell pyroptosis and inflammation in a similar manner (Figure 4(E–H)). Specifically, the inhibiting effects of LncRNA MALAT1 ablation on NLRP3, ASC, IL-1β, and IL-18 expressions in the LPS-treated HK-2 cells (Figure 4(E,F)) and the levels of IL-1β, IL-18, IL-6, and TGF-β in HK-2 cells (Figure 4(G)) and its supernatants (Figure 4(H)) were abolished by co-transfecting cells with miRNA-135b-5p inhibitor.

## Discussion

Recent publications report that AKI is an important risk factor for CKD [42,43], which makes investigations on AKI-associated CKD necessary and meaningful. However, up until now, the therapeutic efficacy of current treatment strategies for AKI-associated CKD is limited as the results of its unclear pathogenesis mechanisms. Hence, it is urgent to uncover the underlying mechanisms that regulate AKI-associated CKD progression. To achieve this, we established LPS-induced AKI models in the human renal tubular epithelial HK-2 cells according to the previous work [8,9]. As expected, LPS suppressed cell proliferation and viability, while promoted cell apoptosis and pro-inflammation cytokines secretion in HK-2 cells, which were supported by the previous literatures [8,9], implying that LPS brought detrimental effects on HK-2 cells . Moreover, consistent with the existed data that cell pyroptosis occurs during the pathogenesis of CKD [44,45] and LPS treatment [46], we evidenced that LPS also triggered NLRP3-mediated pyroptotic cell death in HK-2 cells, suggesting that cell pyroptosis was closely associated with LPS-induced AKI development.

Based on the existed information that LncRNAs play critical roles in regulating AKI and CKD progression [15,16], and LPS-induced cell pyroptosis can be modulated by various LncRNAs, such as LncRNA CTD-2574D22.4 [18] and lncRNA UCA1 [19], we conjectured that there might exist novel LncRNAs that regulated the progression of AKI associated CKD. Among all the LncRNAs, LncRNA MALAT1 involves in regulating inflammation [23–25] and NLRP3-mediated pyroptotic cell death in multiple diseases, including diabetic atherosclerosis [26], and diabetic nephropathy [27]. Interestingly, data from different teams suggest that targeting LncRNA MALAT1 is a novel strategy to attenuate LPS-induced inflammatory responses and AKI [35], LncRNA MALAT1 regulates high-glucose induced cell pyroptosis in endothelial and epithelial cells [21,28], and LncRNA MALAT1 is closely associated with renal ischemia-reperfusion injury and inflammation [47]. In this study, we verified that LncRNA MALAT1 involved in regulating LPS-induced HK-2 cell pyroptosis. Specifically, the expression levels of LncRNA MALAT1 were upregulated in LPS treated HK-2 cells, and we evidenced that the promoting effects of LPS on cell apoptosis, pyroptosis, and inflammation were reversed by silencing LncRNA MALAT1, which were supported by the previous work [21,28,35], indicating that knock-down of LncRNA MALAT1 attenuated LPS-induced HK-2 injury.

According to the principles of LncRNAs-miRNAs-mRNA ceRNA networks [29,30], LncRNAs exert their biological functions through acting as RNA spongers to absorb and restrain the downstream target miRNAs, causing the alterations of expression patterns of the diseases-associated genes. Given that LncRNA MALAT1 positively regulates NLRP3-mediated cell pyroptosis [21,28], we hypothesized that some miRNAs might bridge LncRNA MALAT1 and NLRP3 mRNA. As expected, we screened out a novel miRNA-135b-5p that potentially targeted both LncRNA MALAT1 [37] and exerted its inhibiting effects on NLRP3 [38,39], and further results evidenced that LncRNA MALAT1 positively regulated NLRP3 expressions by sponging miRNA-135b-5p. Interestingly, miRNA-135b-5p reversed LPS induced inflammatory responses [40], and our data supported that LPS downregulated miRNA-135b-5p, and upregulation of miRNA-135b inhibited NLRP3-mediated cell pyroptosis to recover cellular functions in LPS-treated HK-2 cells. In addition, we evidenced that the protective effects of LncRNA MALAT1 ablation on LPS-treated HK-2 cells were abrogated by silencing miRNA-135b-5p, suggesting that LncRNA MALAT1 regulated LPS-induced CKD progression in a miRNA-135b-5p-dependent manner. However, we only conducted *in vitro* experiments to investigate the role of the LncRNA MALAT1/ miRNA-135b-5p axis in regulating LPS-induced AKI, which needed to be further validated by the *in vivo* experiments in our future work.

## Conclusions

Collectively, we concluded that knock-down of LncRNA MALAT1 suppressed NLRP3-mediated pyroptotic cell death *via* releasing miRNA-135b-5p, resulting in the survival of HK-2 cells and targeting the LncRNA MALAT1/ miRNA-135b-5p axis could be used as potential strategy for AKI associated CKD diagnosis and treatment.

## Supplementary Material

Supplemental Material

Supplemental Material

Supplemental Material

Supplemental Material

## Abbreviations

AKI: Acute kidney injury
ceRNA: Competing for endogenous RNA
CKD: Chronic kidney disease
ELISA: Enzyme-linked immunosorbent assay
LncRNAs: Long non-coding RNAs
LPS: Lipopolysaccharide
miRNAs: microRNAs
