# Supplementary figures and images for: LncRNA MALAT1-deficiency restrains lipopolysaccharide (LPS)-induced pyroptotic cell death and inflammation in HK-2 cells by releasing microRNA-135b-5p
Source: Ren Fail. 2021 Sep 9;43(1):1288–97. doi: 10.1080/0886022X.2021.1974037 (PMC8439250; doi:10.1080/0886022X.2021.1974037)

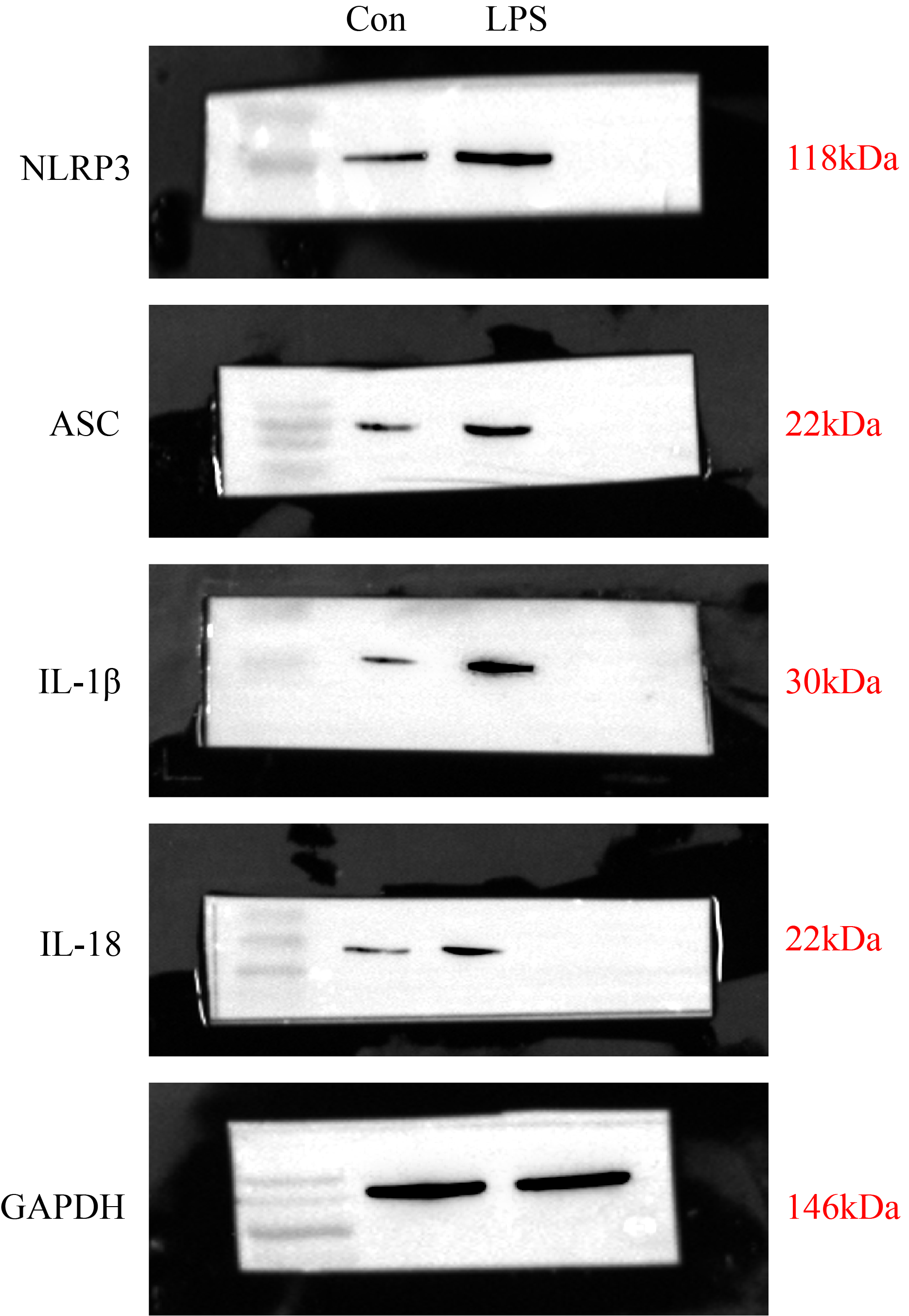

Supplement: Supplemental Material [file IRNF_A_1974037_SM0541.jpg]

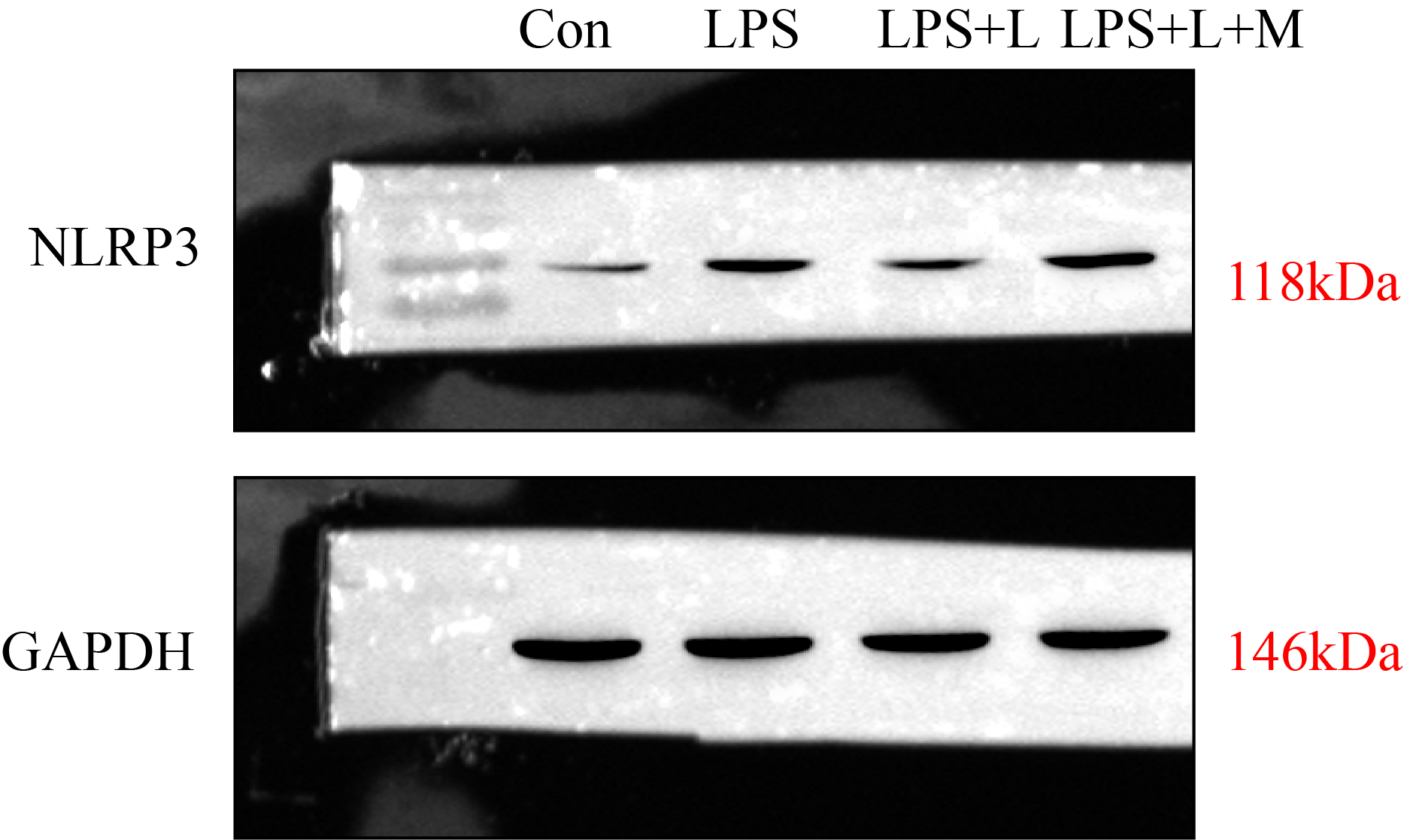

Supplement: Supplemental Material [file IRNF_A_1974037_SM0538.jpg]

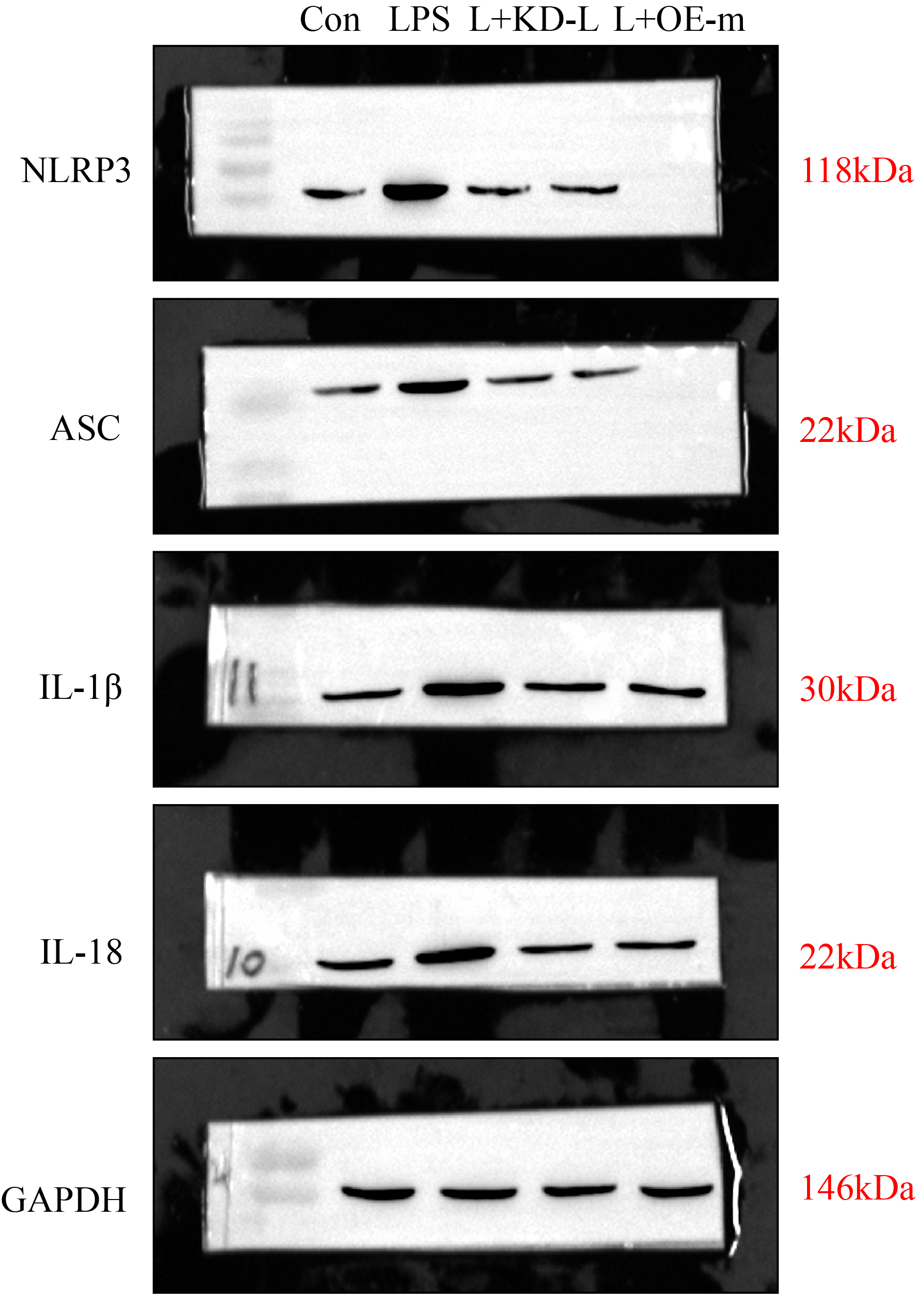

Supplement: Supplemental Material [file IRNF_A_1974037_SM0536.jpg]

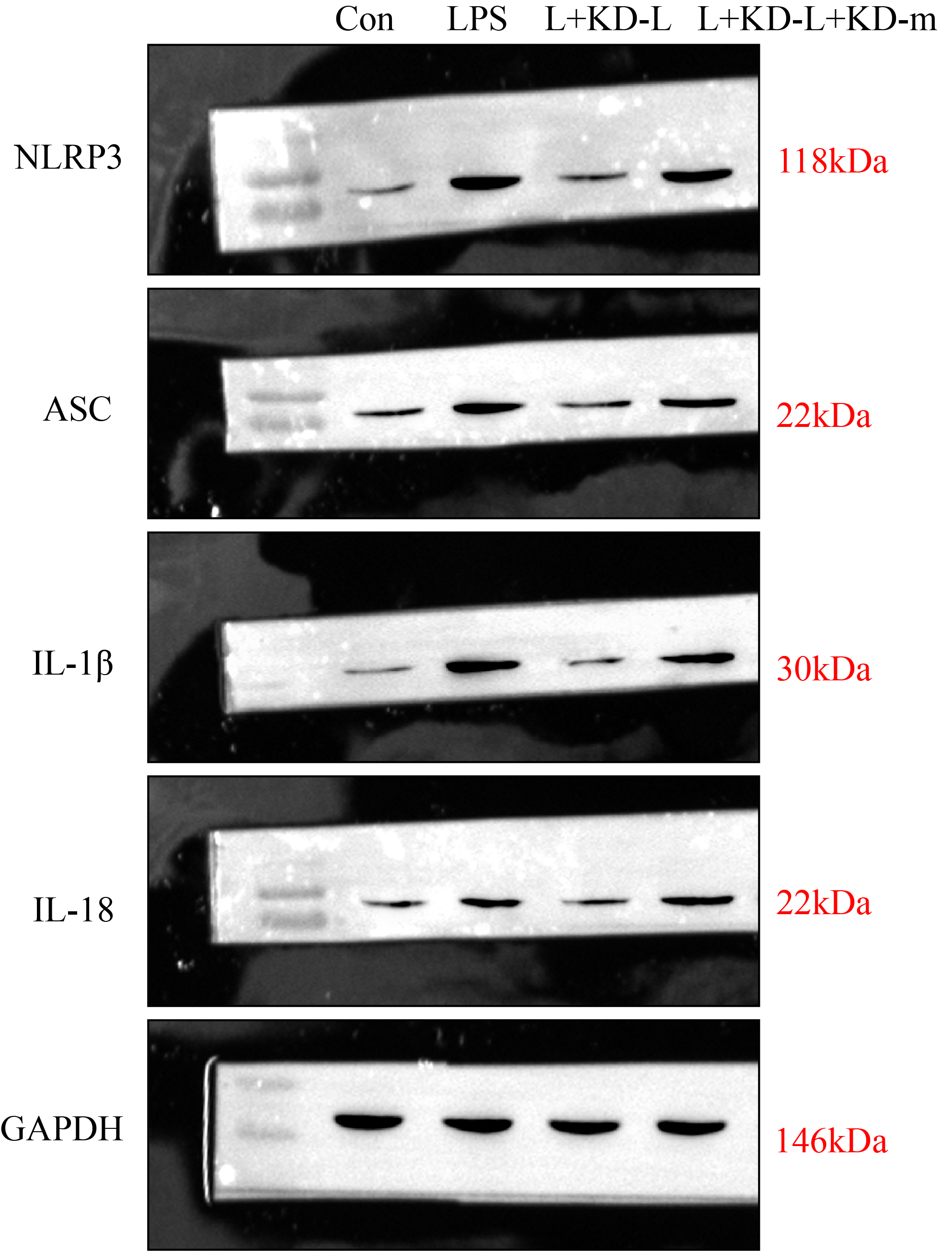

Supplement: Supplemental Material [file IRNF_A_1974037_SM0298.jpg]
